# A Surfactant Concentration Model for the Systematic Determination of the Critical Micellar Concentration and the Transition Width

**DOI:** 10.3390/molecules26175339

**Published:** 2021-09-02

**Authors:** Wajih Al-Soufi, Mercedes Novo

**Affiliations:** Department of Physical Chemistry, Faculty of Science, University of Santiago de Compostela, E-27002 Lugo, Spain; m.novo@usc.es

**Keywords:** surfactants, micelles, critical micelle concentration, concentration model, data analysis

## Abstract

The critical micellar concentration (*cmc*) is a fundamental property of surfactant solutions. Many proposed methods for the definition and determination of the *cmc* from property-concentration plots yield values, which depend on the studied property, on the specific technique used for its analysis and in many cases on the subjective choice of the chosen type of plot and concentration interval. In this focus review, we revise the application of a surfactant concentration model we proposed earlier that defines the *cmc* directly based on the surfactant concentration. Known equations for the concentration-dependence of different surfactant properties can then be combined with this concentration model and fitted to experimental data. This modular concept makes it possible to determine the *cmc* and the transition width in a systematic and unambiguous way. We revise its use in the literature in different contexts: the determination of the *cmc* of surfactants and their mixtures from different properties (electrical conductivity, NMR chemical shift, self-diffusion, surface tension, UV-Vis absorption, fluorescence intensity and fluorescence correlation). We also revise the dependence of the width of the transition region on composition, detailed studies of the properties of fluorescent probes and the aggregation of non-surfactant systems, namely amyloid peptides.

## 1. Introduction

The critical micellar concentration (*cmc*) is a fundamental property of a surfactant solution. At surfactant concentrations near and above the *cmc* the surfactant molecules start to aggregate and form micelles. The formation of micelles affects many properties of a surfactant solution introducing usually a marked change at the *cmc* in plots of a property versus the surfactant concentration. Any property of the surfactant solution affected by the formation of micelles can in principle be used to determine the *cmc*: electrical conductivity of ionic surfactants, surface tension, NMR chemical shift, UV-Vis absorption, fluorescence spectroscopy of a probe molecule, ion-selective electrodes, isothermal titration calorimetry, densimetry, ultrasound spectroscopy, translational diffusion determined by light scattering or fluorescence correlation spectroscopy and many more. Many methods for the definition and determination of the *cmc* from property-concentration plots have been proposed over the last 70 years [1,2,3,4,5,6,7,8,9] and are still published [10,11,12]. Most of these methods determine the *cmc* from the intersection of two straight lines drawn or fitted to the two branches below and above the *cmc*, assuming that the property depends linearly on the concentrations of free surfactant and micelles. In the case of a sigmoidal property- concentration curve, such as those obtained from absorption or fluorescence of a probe, the *cmc* is arbitrarily defined as one of the characteristic points of the curve. Other methods propose to determine the *cmc* from the maxima or zero-crossings of the nth-order derivative of the measured data or of some arbitrary curve fitted to it. These methods yield experimental values of the *cmc*, which depend on the studied property, on the specific technique used for its analysis and in many cases on the subjective choice of the investigator on the type of plot and the concentration interval used.

The methods proposed so far define the *cmc indirectly* as the point of significant change in the measured property, for example a break in the surface tension, in the electrical conductivity of the solution or in the fluorescence emission of a probe molecule. However, the critical micellar concentration is a feature of the surfactant aggregation, and should therefore be defined and analysed directly based on the surfactant concentration. This distinction is relevant for several reasons. Firstly, because a “critical” change in the measured property does not necessarily coincide exactly with the concentration of the critical change at the molecular level. This may seem counterintuitive, but is a direct consequence of the nonlinear dependence of some properties on the concentration. We will revise this effect in more detail in this contribution. Secondly, a *cmc*-definition based on the surfactant concentration itself avoids the ambiguity in its definition based on different properties and procedures.

The big variety of empiric procedures for the determination of the *cmc* from experimental data is probably also due to the complex physicochemical models and numeric simulations of the micellization process [13,14,15,16,17,18,19,20,21,22,23]. Although increasingly sophisticated and precise, these models and simulations are not aimed towards the analysis of experimental data and therefore do not offer fitting functions with the *cmc* as an adjustable parameter that could be fitted to experimental data. Of course, it would be desirable to derive such a function from theoretical principles.

Nearly ten years ago we proposed a surfactant concentration model, which we derived from established empiric features of surfactant solutions [9]. This model allows one to calculate the concentrations of monomeric and micellized surfactants in solution for a given total surfactant concentration. It depends on only two adjustable parameters, the *cmc* and the *width of the transition region* around the *cmc*.

We then combined this concentration model with the known equations for the concentration dependence of several properties of surfactant solutions, specifically with those of electrical conductivity, NMR chemical shift, self-diffusion, surface tension, UV-Vis absorption, steady-state and time resolved fluorescence intensity and fluorescence correlation [9,24,25]. The resulting set of equations for the concentration dependence of the properties proved to fit excellently the experimental data and gave consistent values among the properties of the *cmc* and of the transition width.

The separation between the concentration model and the experimental property has many advantages. The *cmc* is now directly a feature of the surfactant concentration and is common to all derived properties. Its value can be unambiguously and precisely determined from fits to experimental data, even by simultaneous (global) fits of data from different techniques.

Another advantage is that, once we prove that the concentration model is sufficiently precise for a given surfactant system, we can test the validity and the limitations of the equations describing the concentration dependence of the solution property.

The objective definition of the *cmc* as part of the concentration model and its determination from data regression also avoids errors inherent to subjective graphical procedures.

Finally, the model we proposed also introduces naturally the transition region between purely monomeric and dominantly micellized surfactant. It is known that micelles are already formed at concentrations below the *cmc* and there is some controversy whether these pre-micelles should be considered as proper species [23,26,27].

In this focus review, we summarize shortly the surfactant concentration model and its application to different properties. Then we revise its use in the literature in different contexts: the determination of the *cmc* of surfactants and their mixtures, as basis of theoretical models or new derived properties, the discussion of the width of the transition region, detailed studies of the properties of fluorescent probes and the aggregation of non-surfactant systems, namely peptides.

This work is divided into the following sections: (1) Introduction; (2) The Surfactant Concentration Model; (3) Application to Different Techniques; (4) Influence of Nonlinear Property-Concentration Relationships on the *cmc* Value; (5) Analysis with Sparse Data Points; (6) Conclusions. Examples of the application of the concentration model to several surfactant systems and properties is presented of referenced: conductivity data of the anionic surfactant SDS (Section 3.1.1 and Section 5), of mixtures of two cationic surfactants, tetradecyltrimethylammonium bromide (TTAB), and methylammonium dodecylethylmethacrylate bromide (Section 3.1.2), of two antidepressant surface-active drugs, imipramine (IMP) and clomipramine (ClMP) hydrochloride (Section 3.1.3). Surface tension of a small molecule hydrogelator with surfactant-like properties, Fmoc-phenylalanine (Section 3.2), and of the mixture of the anionic surfactant, dodecylbenzene sulphonic acid (DBSA) and poly (ethylene glycol) (PEG) (Section 3.2). NMR-Chemical shift of two cationic (CTAB, TTAB), one anionic (SDS), and a nonionic surfactant (Triton X-100) (Section 3.3). Fluorescence of the probes perylene, pyrene, Coumarine 153 (C153) and 2-anilinonaphthalene-6-sulfonic acid (ANS) in Triton X-100 (Section 3.4). The spectral ratios I/III of pyrene in TX100 and SDS (Section 3.4.1). UV-Vis absorbance of Triton X-100 (Section 3.5). Fluorescence Correlation Spectroscopy (FCS) of Rhodamine 123, Coumarine 152 and BODIPY-labelled cholesterol in the presence of Triton X-100 (Section 3.6) and of Amyloid-β(1–42) peptides (Section 3.7). Conductivity and density measurements of SDS and sodium lauryl ether sulfate (SLES) (Section 3.8). The dependence of the ZnS nanoparticle radius on the CTAB concentration (Section 4).

## 2. The Surfactant Concentration Model

We derived the model in detail before [9]. Here we will summarize its main properties. The starting point for the derivation of the concentration model is the definition of the *cmc* itself. Already in 1955 Phillips proposed to define the *cmc* as “the point corresponding to the maximum change in gradient in an ideal property-concentration relationship” [1]. This so-called “Phillips-condition” has been extensively used in the literature with more than 650 citations so far. It is an empiric condition, not derived from a more fundamental theory of micellization. As discussed by Rusanov, several other analogue conditions could also be used [28].

The Phillips condition refers originally to the change in the “property-concentration relationship”, whereas we applied the condition directly to the surfactant concentration. We define the *cmc* as the total surfactant concentration [S]_0_ at which the change in the gradient of the monomer concentration [S_1_] with respect to [S]_0_ is maximal, that is where the third derivative of [S_1_] is zero:

(1) $${\left(\frac{d^{3}[S_{1}]}{d{\left[S\right]}_{0}}\right)}_{{\left[S\right]}_{0}=cmc}\;=0$$

In the same way, we transfer the concepts developed by García-Mateos et al. [3] for conductivity data directly to the surfactant concentration. They applied a probabilistic point of view to the pseudophase separation model and interpret the distribution of counterions or of monomers in the aqueous and micellar phases with the aid of a binomial distribution model between micellized or free states. The degree of micellization represents then the distribution function, which becomes a continuous normal distribution when the number of micellized surfactants is high enough (central limit theorem).

After some substitutions and normalisation, they show that the second derivative of the electrical conductivity with respect to the surfactant concentration is a Gaussian function centred at the *cmc* with some width *σ*. They fit conductivity data with a double numerical integration of this Gaussian function.

**Figure 1:**
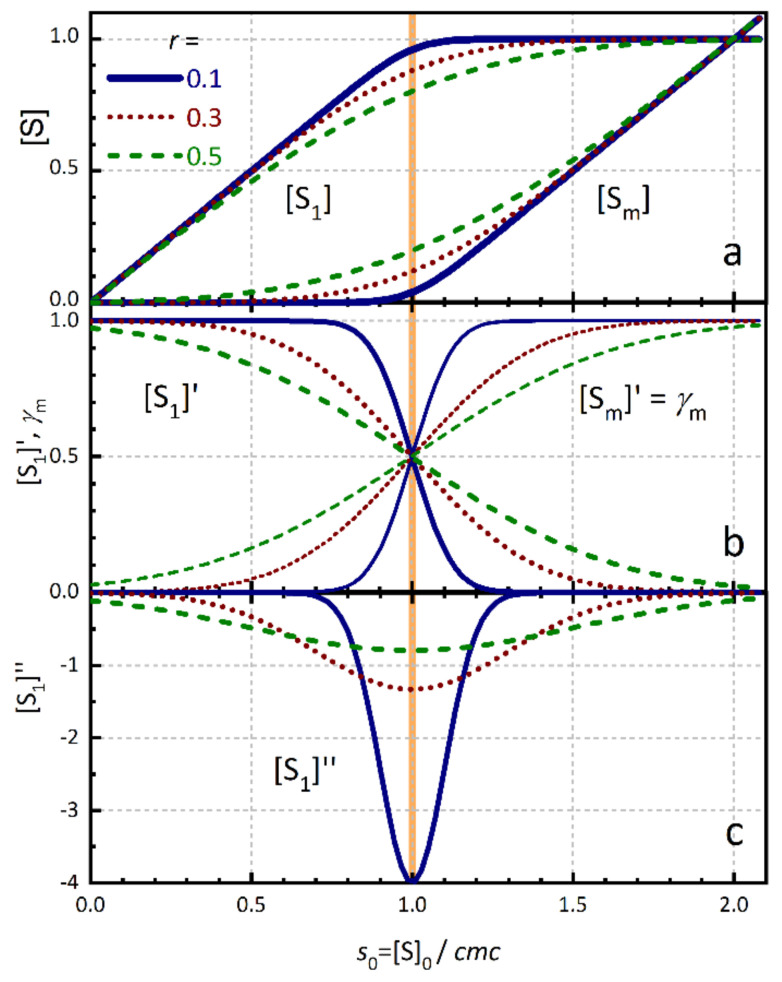
Surfactant concentration model. (**a**) Concentration of monomeric surfactant [S_1_] and of micellized surfactant [S_m_] as function of the relative total surfactant concentration *s*_0_ = [S]_0_/*cmc* (Equations (5)–(8)) for three values of the relative transition width *r* (solid: *r* = 0.1, dot: *r* = 0.3, dash: *r* = 0.5). (**b**) First derivative [S_1_]′ = d[S_1_]/d[S]_0_ (Equation (9)) and (differential) degree of micellization γ_m =_ [S_m_]′ = d[S_m_]/d[S]_0_ (Equation (10)) (**c**) Second derivative [S_1_]″ = d^2^[S_1_]/d[S]_0_^2^ (Equation (2)). The vertical thick line indicates the *cmc*. (Adapted with permission from [9]).

We apply this concept to the surfactant concentration. We describe the second derivative of [S_1_] with respect to [S]_0_ by a Gaussian function centred at the *cmc*, with amplitude *A* and width *σ* as given in Equation (2) (Figure 1c):

(2) $$\frac{d^{2}{[S}_{1}]}{d{\left[S\right]}_{0}^{2}}=\frac{-A}{\sqrt{2\pi }\sigma }e^{-\frac{{\left({\left[S\right]}_{0}-cmc\right)}^{2}}{2\sigma^{2}}}$$

The standard deviation *σ* of the Gaussian (half width at $e^{-1/2}$) is a measure of the width of the transition region around the *cmc*. In this region, *cmc ± σ*, the concentration of surfactant monomers still increases with [S]_0_, but first micelles are already formed. The smaller *σ*, the sharper is the transition between the two linear regions below and above the *cmc*.

In order to facilitate the comparison of transition widths *σ* of surfactants with different *cmc* values, we define the relative transition width *r*:

(3) r=σ/cmc

Ideally, below the *cmc*, the concentration of monomeric surfactant [S_1_] is equal to the total surfactant concentration [S]_0_ and stabilizes at the value of the *cmc* itself:

(4) $$[S_{1}]=\left\{\begin{array}{c} {\left[S\right]}_{0,}\\ cmc, \end{array}\begin{array}{c} \;{\left[S\right]}_{0}<<cmc\\ \;{\left[S\right]}_{0}>>cmc \end{array}\right.$$

The concentration of micellized surfactant, [S_m_], is determined from the difference between the total concentration and that of monomeric surfactants:

(5) $$\left[S_{m}]={\left[S\right]}_{0}-[S_{1}\right]$$

The concentration of micelles, [M], depends on the mean aggregation number *n*:

(6) $$[M]=[S_{m}]/n$$

After double algebraic integration of the Gaussian of Equation (2) and proper normalization using Equations (1)–(6) we obtain the central equation of the concentration model, the monomer concentration [S_1_] as a function of the total surfactant concentration [S]_0_ (Figure 1a) [9]:

(7) $$\begin{array}{l} {}[S_{1}]=cmc-\frac{A}{2}\left(\sqrt{\frac{2}{\pi }}\;\sigma \;e^{-\frac{{\left({\left[S\right]}_{0}-cmc\right)}^{2}}{2\sigma^{2}}}+({\left[S\right]}_{0}-cmc)\left(\mathrm{erf}\left(\frac{{\left[S\right]}_{0}-cmc}{\sqrt{2}\sigma }\right)-1\right)\right)\\ =cmc\left[1-\frac{A}{2}\left(\sqrt{\frac{2}{\pi }}\;r\;e^{-\frac{{\left(s_{0}-1\right)}^{2}}{2r^{2}}}+(s_{0}-1)\left(\mathrm{erf}\left(\frac{s_{0}-1}{\sqrt{2}r}\right)-1\right)\right)\right] \end{array}$$

with the relative total surfactant concentration $s_{0}={\left[S\right]}_{0}/cmc$ and the amplitude *A* (For *r* < 0.4 the amplitude *A* ≈ 1):

(8) $$A=\;\frac{2}{1+\sqrt{\frac{2}{\pi }}\;r\;e^{-\frac{1}{2r^{2}}}+\mathrm{erf}\left(\frac{1}{\sqrt{2}\;r}\right)}$$

This model assumes a constant aggregation number *n*, also at the onset of micelle formation at the *cmc*. This is in line with simulations [29] and thermodynamic models [20] and proved to describe well the behaviour of several surfactants [9].

The surfactant concentration model, Equations (5)–(8), can be plugged into equations that describe derived properties obtained with different techniques. The resulting equations for the property-concentration relationships can be easily implemented as fitting functions in analysis software. Functions for OriginPro (OriginLab Corporation, Northampton, MA, USA) and other software packages can be downloaded from the webpage of the authors.

The first derivative [S_1_]′ of the monomer concentration of Equation (7) is given by the error function erf(*x*), which is a sigmoid type function centred at *x*=0 (in this case at the *cmc*) with limiting values $\mathrm{erf}\left(0\right)=0$, erf(−∞)=−1, and erf(∞)=1 (Figure 1b).

(9) $$\frac{d[S_{1}]}{d{\left[S\right]}_{0}}=\frac{A}{2}\left(1-\mathrm{erf}\left(\frac{{\left[S\right]}_{0}-cmc}{\sqrt{2}\sigma }\right)\right)$$

[S_1_]′ is directly related to the differential degree of micellization *γ*:

(10) $$\gamma =\frac{d[S_{m}]}{d{\left[S\right]}_{0}}=1-\frac{d[S_{1}]}{d{\left[S\right]}_{0}}$$

The second derivative [S_1_]″ is of course the Gaussian function of Equation (2) (Figure 1c).

The value of the (fractional) degree of micellization α_m_ = [S_m_]/[S]_0_ (or molar fraction of micellized surfactant *X*_m_ = α_m_) at the *cmc* is given approximately by:

(11) $$\alpha_{m}(cmc)={\left(\frac{\left[S_{m}\right]}{{\left[S\right]}_{0}}\right)}_{cmc}\approx \frac{r}{\sqrt{2\pi }}\approx 0.4\times r$$

For a typical value of *r* = 0.1 only 4% of the surfactant molecules form micelles at the *cmc*.

## 3. Application to Different Techniques

### 3.1. Electrical Conductivity of Ionic Surfactant Solutions

Data of the conductivity (specific conductance) κ of a solution of an ionic surfactant of concentration [S]_0_ can be fitted with the linear model [9,30]

(12) $$\begin{array}{l} \kappa =a[S_{1}]+b[S_{m}]+\kappa_{s}\\ \;\;=a[S_{1}]+b({\left[S\right]}_{0}-[S_{1}])+\kappa_{s} \end{array}$$

The parameters *a* and *b* have units of molar conductivity and are the slopes of the limiting straight lines $\kappa_{1}=a{\left[S\right]}_{0}+\kappa_{s}$ and $\kappa_{2}=a\cdot cmc+b\cdot ({\left[S\right]}_{0}-cmc)+\kappa_{s}$ observed at low and high concentrations of surfactant, respectively [9]. $\kappa_{s}$ is the residual conductivity of the solvent without surfactant. The concentration [S_1_] is calculated from [S]_0_ using Equation (7) of the concentration model.

The molar conductivity (equivalent conductance) $\Lambda_{m}$ is given by:

(13) $$\Lambda_{m}=\left(\kappa -\kappa_{s}\right)/{\left[S\right]}_{0}=a\left[S_{1}\right]/{\left[S\right]}_{0}+b\left[S_{m}\right]/{\left[S\right]}_{0}$$

This model reproduces conductivity data of SDS, DTAB, CTAB and LAS very well, and extracts precise *cmc* values even in cases with very small change in the slope below and above the *cmc* [9].

#### 3.1.1. Comparison to the Carpena Model

The method proposed by Carpena et al. [6] pursued the same aim as our concentration model, to eliminate the imprecision and subjectivity inherent to the determination of the *cmc* by graphical methods. Their method is specific for conductivity data and clearly inspired the more general approach we proposed ten years later.

The authors observed that experimental data of the differential conductivity $\Lambda_{diff}=d\kappa /d{\left[S\right]}_{0}$ are well fitted by a Boltzmann type sigmoidal centred at the *cmc* with the transition width Δ[S]_0_:

(14) $$\Lambda_{diff\;Carpena}=d\kappa /d{\left[S\right]}_{0}=\frac{a-b}{1+e^{\left({\left[S\right]}_{0}-cmc\right)/\Delta {\left[S\right]}_{0}}}+b$$

Here we use the same nomenclature as in Equation (13). Integration of this sigmoidal and proper normalisation leads to an expression of the conductivity as a function of [S]_0_:

(15) $$\kappa_{Carpena}=\kappa_{s}+a{\left[S\right]}_{0}+\Delta {\left[S\right]}_{0}(b-a)\ln\left(\frac{1+e^{\left({\left[S\right]}_{0}-cmc\right)/\Delta {\left[S\right]}_{0}}}{1+e^{-cmc/\Delta {\left[S\right]}_{0}}}\right)$$

This expression can be fitted to conductivity data in order to obtain the *cmc* and the width of the transition Δ[S]_0_. The authors and others applied this method successfully to a variety of ionic surfactant systems.

How does this method compare to the concentration model (Equations (7) and (12)) we proposed?

(i) Both methods are based on empirical models. The concentration model relies on the Phillips condition for the *cmc* and on the description of the degree of micellization as a continuous normal distribution function, derived from a probabilistic description of the distribution of free and micellized surfactants. The Carpena method starts with the Boltzmann function for the description of the differential conductivity. Other conditions or sigmoidal functions would lead to other models with similar results. However, the concentration model is derived from more general assumptions that make it applicable to any property-concentration relationship.
(ii) The value of the transition width Δ[S]_0_ of the Carpena method, has no direct relation to that of the transition width *σ* = *r*·*cmc* of the concentration model, which stems from the width of the Gaussian function of Equation (2). Both widths define the slopes of the corresponding sigmoidal functions at the inflection point [S]_0_ = *cmc*:
  (16) $$\frac{d^{2}\kappa }{d{\left[S\right]}_{0}^{2}}{\left(cmc\right)}_{Carpena}=\frac{b-a}{4\Delta {\left[S\right]}_{0}}\;\frac{d^{2}\kappa }{d{\left[S\right]}_{0}^{2}}(cmc)\approx \frac{b-a}{\sqrt{2\pi }\sigma },\;\;(r<0.4,A\approx 1)$$

The ratio $\sigma /\Delta {\left[S\right]}_{0}\approx 4/\sqrt{2\pi }=1.60$ yields sigmoids with the same slope at the *cmc*. The best fit between both curves is obtained with a ratio $\sigma /\Delta {\left[S\right]}_{0}$= 1.75. In practice, fits of conductivity data with both methods yield transition width with ratios $\sigma /\Delta {\left[S\right]}_{0}$ between 1.5 and 1.8.

(iii) The values of the *cmc* determined by the two methods are in practice identical. The sigmoidal Boltzmann function of Equation (14) is not identical to the sigmoidal error function of Equation (9), but very similar. The fit of the SDS conductivity data of our original paper (Figure 2 in [9]) with Equation (15) yields *cmc_Carpena_* = 8.096 ± 0.005 mM, identical to *cmc* = 8.099 ± 0.005 mM obtained with Equation (12). The same is true for the slopes *a* and *b*. The reduced *χ*^2^ values are very similar with *χ*^2^ = 0.436 and *χ*^2^ = 0.427 for Equations (12) and (15), respectively. The transition width is Δ[S]_0_ = 0.519 mM. Compared to the transition width from the concentration model *σ* = *r·cmc* = 0.112 × 8.099 mM = 0.907 mM, the ratio *σ*/Δ[S]_0_ = 1.75 falls within the expected interval indicated above.

#### 3.1.2. Example of the Use for Surfactant Mixtures

Pereyra et al. [31] studied mixtures of two cationic surfactants, a conventional one, tetradecyltrimethylammonium bromide (TTAB), and a polymerizable surfactant (PS), methylammonium dodecylethylmethacrylate bromide. They analyzed conductivity data of the mixtures using three methods, the excess specific conductivity method, the Carpena method discussed above [6], and the concentration model (Equations (7) and (12)).

The *cmc* values they obtain from fits with our surfactant model (Equation (12)) are virtually identical to those from fits with the Carpena method for all mixtures.

The excess specific conductivity method depends on a graphical determination of the intersection of two straight lines. It yields *cmc* values, which coincide well with those from the fits at low mole fraction of PS, but not at higher ones. This is probably due to the limitations of the graphical method. At high PS content, the *cmc* lies at the upper end of the studied surfactant concentration interval. This, together with the increase of the transition width, makes the graphical determination of the limiting line very unreliable. The fit of the full curve with one of the models, our model or Carpena’s, is much less affected by this problem.

The authors also analyse the variation of the transition width with composition. The relative transition width *r* of the TTAB-PS mixtures increases with increasing mole fraction of PS, from *r* = 0.11 for pure TTAB up to *r* = 0.33 for pure PS (Figure A2). Interestingly, the variation of *r* with α_PS_ is well fitted with Clint’s model derived for the *cmc* of surfactant mixtures (Figure A2).

The ratio *σ*/Δ[S]_0_ = 1.58–1.73 of the transition width determined from the two methods is not constant but falls within the expected interval (see above).

The plots of the Gaussian functions (Equation (2)) for the different mixtures the authors show seem to be a very good way to illustrate the change in the transition width. These Gaussians can be easily calculated with the functions we provide.

The authors also compare the concentrations of free and bound counterions, determined in ion-selective-electrode (ISE) experiments, with those calculated with the concentration model Equations (7) and (12). They find good agreement at low PS content but deviations at PS rich mixtures. It will be definitely worth to analyse these results in more detail and to extend the concentration model to ISE data.

#### 3.1.3. Global Fit of Conductivity Data. Application to Weakly Surface-Active Drugs

One of the advantages of the concentration model (7) and (12) over graphical or procedural methods is the possibility to perform global analysis, sharing some of the parameters of the model among the fits to all curves of a series, for example among conductivity titration curves measured at different temperatures. Global fits of a model to series of data reduces the number of fitting parameters and thus the effect of parameter correlation [31,32,33,34,35,36].

Global fitting helps to extract the *cmc* and the transition width even for surfactants presenting only a very small change in the slopes of their conductivity-concentration plots and wide transition intervals.

**Figure 2:**
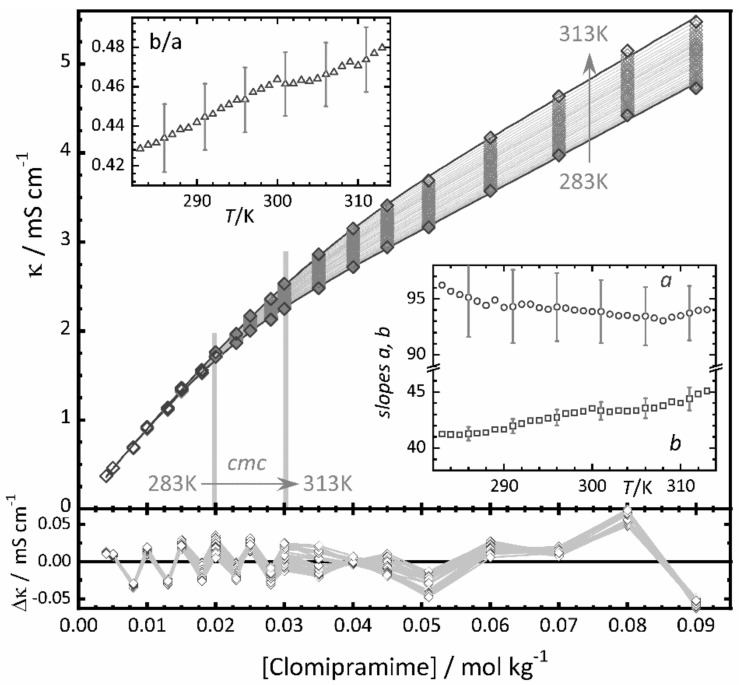
Conductivity of clomipramine hydrochloride (ClMP) in pure water as function of molal concentration and temperature. Black and grey diamonds: experimental data [37]. Grey and black lines: global fit of Equations (7) and (12) with *r* = 0.53 ± 0.04, the parameters indicated in the insets, and the *cmc* shown in Figure 3. Vertical grey lines: range of *cmc* values obtained from the fit. Right Inset: slopes *a* and *b* from the fit in units of mS cm^−1^ kg mol^−1^. Left Inset: slope ratio *b*/*a*. Lower panel: residuals of the fit.

**Figure 3:**
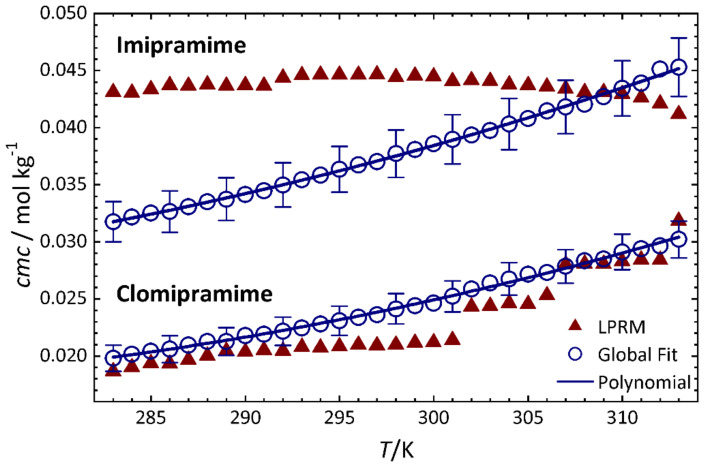
Values of the *cmc* of IMP and ClMP at different temperatures obtained by different analysis methods from the data of Figure 2 and Figure A1. Blue circles: from global fits of Equation (12) with shared *r*. Red triangles: from LPRM [37]. The continuous line represents a second order polynomial fit through the values from the global fit (Table A1).

**Figure A1:**
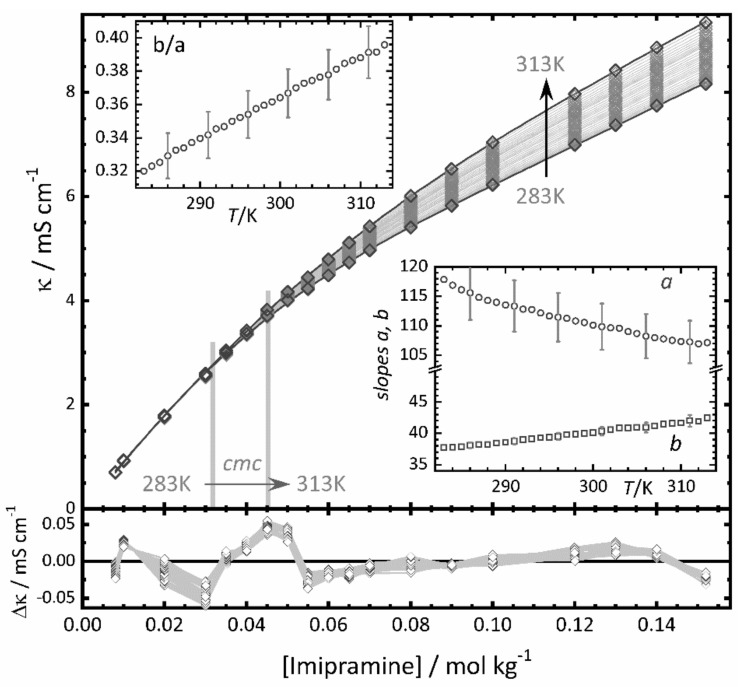
Conductivity of imipramine hydrochloride (IMP) in pure water as function of molal concentration and temperature. Black and grey diamonds: experimental data [37]. Grey and black lines: global fit of Equations (7) and (12) with *r* = 0.94 ± 0.08, the parameters indicated in the insets, and the *cmc* shown in Figure 3. Vertical grey lines: range of *cmc* values obtained from the fit. Right Inset: slopes *a* and *b* from the fit in units of mS cm^−1^ kg mol^−1^. Left Inset: slope ratio *b*/*a*. Lower panel: residuals of the fit.

**Figure A2:**
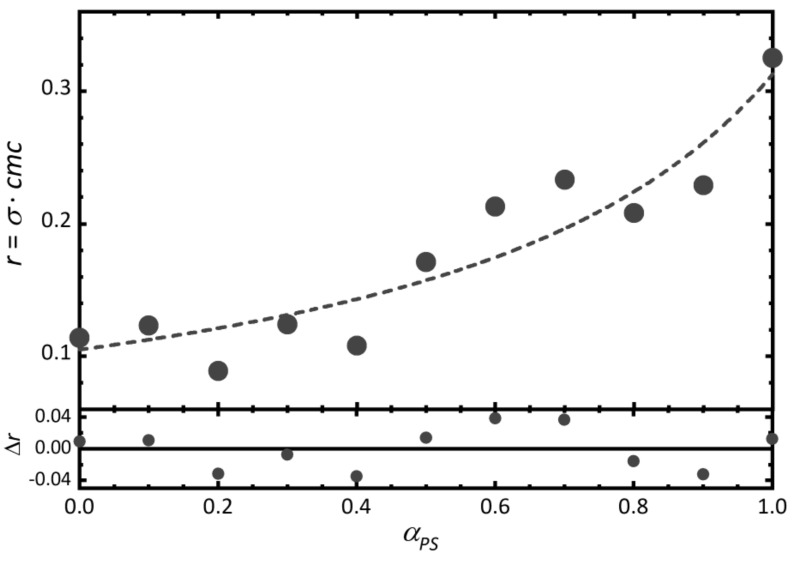
Relative transition width *r* as function of the mole ratio α_PS_ of mixtures of TTAB and PS reported by Pereyra et al. [31]. The dashed line is a fit of Clint’s model *r* = 1/[(1 − α_PS_)/*r_TTAB_* + α_PS_/*r_PS_*] to the data with *r_TTAB_* = 0.105 and *r_PS_* = 0.31.

In order to illustrate global fitting we use electrical conductivity data published by López Fontán et al. of aqueous solutions of two antidepressant surface-active drugs, imipramine (IMP) and clomipramine (ClMP) hydrochloride. The data set consists of titrations at 19 concentrations and 31 temperatures between 283 K and 313 K [37]. Figure 2 shows the data for ClMP (diamonds) and Figure A1 that of IMP. The determination of the *cmc* is difficult due to the small change in the slopes and the very wide transition interval, especially in the case of IMP. The authors estimated the *cmc* values from the maxima of the second order derivatives of the conductivity data with a local polynomial regression method (LPRM) based on nonparametric regression [8]. The authors do not indicate uncertainties. Their *cmc* values (red triangles in Figure 3) show discontinuities within each series and, importantly, opposite temperature dependencies. In the case of IMP the temperature dependence is slightly convex with an overall decrease in the *cmc* with increasing temperature, whereas for ClMP a steady increase is obtained.

Individual fits of the concentration model (Equation (12)) to each of the curves are excellent at all temperatures for both drugs. However, at high temperatures and especially in the case of IMP the uncertainty of the determination of *cmc* and *r* from these individual fits is huge, leading to unreliable results. We observed, though, that the values of the relative transition width *r* do not significantly change within their errors with temperature, and that global fits of Equation (12) with a shared value of *r* yield very similar residual patterns and practically the same value of the reduced *χ*^2^ as the individual fits. This justifies the application of global fits, assuming a temperature independent transition width *r*.

Figure 2 shows the results of the global fit of Equation (12) to the whole dataset of ClMP and Figure A1 to that of IMP. The fit was performed with a commercial software (Origin Pro). The black and grey diamonds are the experimental data values. The thin grey lines are the fit curves. The lower panels in the figures indicate the residual errors.

The temperature dependence of the *cmc* values (blue circles in Figure 3) shows now no discontinuities and is very similar for both drugs. The *cmc* values are relatively high (24 ± 1 × 10^−3^ mol kg^−1^ for ClMP and 38 ± 2 × 10^−3^ mol kg^−1^ for IMP, both at 298 K) and increase about 10 × 10^−3^ mol kg^−1^ in the studied 30 K temperature interval. The fit of a second order polynomial gives nearly identical values for both drugs, except for a constant offset (See Figure 2 and Table A1 in the Appendix A).

The values of the slopes *a* and *b* obtained from the global fit are plotted as insets in Figure 2 together with the ratio *b*/*a* = 0.41–0.48. The increase in temperature causes a decrease of *a* but an increase *b*, resulting in a strong rise of the ratio *b*/*a*.

The relative transition widths of the two drugs do not depend on the temperature and are very wide (ClMP: *r* = 0.53 ± 0.04, IMP: 0.94 ± 0.08). This is in line with the approximate rule that the transition width increases with increasing *cmc*, both in absolute terms as well as relative to the *cmc* [2].

### 3.2. Surface Tension

Surface tension (ST) is one of the most frequently used property for the determination of the *cmc*, especially in the case of nonionic surfactants.

The Szyszkowski equation describes approximately the dependence of ST on the surfactant concentration:

(17) $$\gamma =\gamma_{0}-a\ln(1+K_{ad}\cdot [S_{1}])$$

with the adsorption equilibrium constant *K_ad_*, the surface tension of the solvent $\gamma_{0}$, and the constant a=R·T/ω, ω being the cross sectional area of the surfactant molecule at the surface per mol [38,39,40]. This equation depends only on the monomer concentration [S_1_], as micelles are not surface active. [S_1_] is calculated from the concentration model, Equation (7).

The Szyszkowski Equation (17), in combination with the concentration model Equation (7), was used by several authors for the *cmc*-determination from ST data.

The *cmc* values obtained with the concentration model from ST and from NMR spectroscopy data of fluorinated surfactants agree within the experimental error with the results from ITC, dynamic light scattering, small-angle X-ray scattering, and analytical ultracentrifugation [41,42,43].

Gahane et al. tested the antibacterial activity against gram-positive bacteria in gel and solution phases of Fmoc-phenylalanine (Fmoc-F), a small molecule hydrogelator with surfactant-like properties and a *cmc* near its minimum bacteriocidal concentration [44]. They found that its antibacterial effect correlates linearly with its surfactant properties. The authors determined the ST of the Fmoc-F solution using a contact angle goniometer and by the drop count method using a stalagmometer. They fitted the ST data of both methods with Equations (7) and (17) with fixed values of *a* and *r*. The authors only reported the *cmc* value from the distorted stalagmometer data, whereas the cleaner goniometer curve seems to lead to a slightly higher *cmc*. In both cases, the concentration model allows them to extract a *cmc* value from curves with very few data points at concentrations above the *cmc*.

Artykulnyi et al. studied the changes in the structure and interaction of micelles of an anionic surfactant, dodecylbenzene sulphonic acid (DBSA), in aqueous solutions upon addition of a neutral polymer, poly (ethylene glycol) (PEG) [45]. Their surface tension data showed the formation of surfactant-polymer complexes, which lead to a shift of the *cmc* as compared to non-modified surfactant solutions and to the appearance of a critical aggregation concentration (*cac*) at lower concentrations than the *cmc*. The authors make extensive use of Equations (7) and (17) for the determination of both the *cmc* and the *cac* from the same ST-curve, selecting manually the fitting data regions around the critical concentrations.

### 3.3. NMR-Chemical Shift

The chemical shift $\delta_{obs}$ of the resonance peak of the surfactant observed in NMR spectra can be expressed as weighted mean of the chemical shifts $\delta_{1}$ and $\delta_{m}$ of monomeric and micellized surfactant, respectively [9,46]

(18) $$\delta_{obs}=\delta_{1}\cdot \frac{\left[S_{1}\right]}{{\left[S\right]}_{0}}+\delta_{m}\cdot \frac{\left[S_{m}\right]}{{\left[S\right]}_{0}}=(\delta_{1}-\delta_{m})\cdot \frac{\left[S_{1}\right]}{{\left[S\right]}_{0}}+\delta_{m}$$

The concentration [S_1_] is determined by Equation (7). The shifts of several nuclei should be fitted globally.

Yu et al. analysed the ^1^H chemical shifts of two cationic (CTAB, TTAB), one anionic (SDS), and a nonionic surfactant (TX100), both with Equation (18) and with the graphical method of intersecting straight lines and found good coincidence [47]. However, the rather surprising, apparent dependence of the *cmc* values on the position of the proton in the surfactant molecules may as well be an artefact due to the uncertainty in their *cmc* determination. The nonlinear fits of the concentration model yield not only the best estimate of the *cmc* but also the uncertainty of the estimation. Reporting the error intervals of the *cmc* values would have allowed one to assess the statistical significance of the variation they found.

As mentioned before, in a series of publications, Durand and coworkers, used Equation (18) to analyse ^19^F NMR-shift data and obtained good coincidence in the *cmc* values obtained with several other techniques [41,42,43].

### 3.4. Fluorescence of a Dye Probe Molecule

Many fluorescent probe molecules are sensitive to their environment and change their emission properties (intensity, spectral shift, band ratio, lifetime, anisotropy) upon inclusion into a micelle. Hydrophobic dyes with a preferential solubilisation in micelles are widely used for the characterisation of surfactant solutions [7,48,49,50,51]. However, even hydrophobic dye molecules constantly exchange to some extent between the aqueous and the micellar environments. The molar fractions of free and bound dye depend on the binding equilibrium constant and on the micelle concentration [24]. With the aid of the concentration model, we could show that, for a precise determination of the *cmc*, it is imperative to take the dye exchange equilibrium into account. It is generally not correct to assume that all dye molecules are exclusively located within micelles, especially not at concentrations immediately above the *cmc*.

The intensity of the fluorescence emission of a dye undergoing an exchange equilibrium (Equation (19)) is given by the sum of the emissions of free (*D_f_*) and bound dye (*D_b_*) as given in Equation (20). $F_{f}(\lambda )$ and $F_{b}(\lambda )$ are the respective limiting fluorescence intensities, *K* is the binding equilibrium constant, and *X_f_* and *X_b_* are the molar fractions of the free and the bound dye, respectively. The micelle concentration [M] is calculated with the concentration model, Equations (5)–(8).

(19) $$D_{f}+M\overset{K}{⇌}D_{b}\;K=\frac{\left[D_{b}\right]}{\left[D_{f}][M\right]}$$

(20) $$F(\lambda ,{\left[S\right]}_{0})=F_{f}(\lambda )\cdot X_{f}+F_{b}(\lambda )\cdot X_{b}=\frac{F_{f}(\lambda )}{1+K\cdot [M]}+\frac{F_{b}(\lambda )\cdot K\cdot [M]}{1+K\cdot [M]}$$

**Figure 4:**
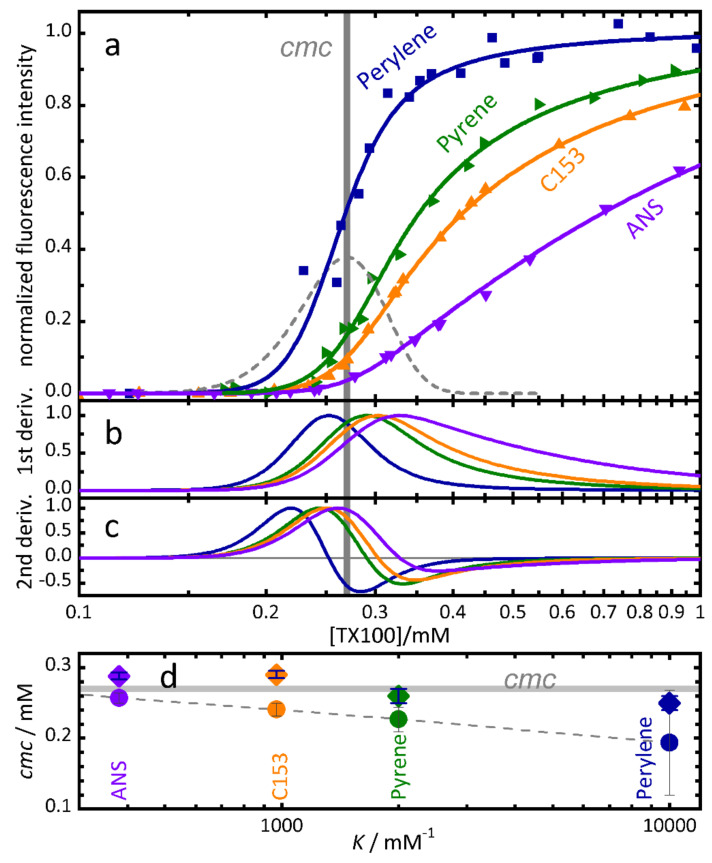
(**a**) Symbols: normalized fluorescence emission intensity of dyes with increasing hydrophobicity in aqueous solutions of TX100 as function of surfactant concentration. Continuous curves: global fit of Equations (5)–(8) and (20) with *cmc* = 0.27 mM, *r* = 0.15. For details see [24]. Dashed line: second derivative of [S_1_] indicating the transition region. Note the logarithmic concentration scale. (**b**) First derivatives of the fluorescence intensities of panel (**a**). (**c**) Second derivatives of the fluorescence intensities of panel (**a**). (**d**) *cmc*-values estimated with two methods versus the binding constant *K* of the dye. Diamonds: *cmc* values determined from individual fits of the concentration model Equations (5)–(8) and (20) to the fluorescence emission intensities of panel (**a**). Filled circles: *cmc* values determined from the traditional graphical method based on the intersection of straight lines. Grey horizontal line: *cmc* = 0.27 mM estimated from direct TX100 absorbance. (Adapted with permission from [24]).

Figure 4 shows the normalized fluorescence emission intensity of several typical fluorescent probes with increasing hydrophobicity in aqueous solutions of TX100 as a function of surfactant concentration. The *cmc* values determined with the graphical method of intersecting straight lines depend significantly on the binding equilibrium constant between dye and micelles (Filled circles Figure 4d). In contrast, fits with the concentration model, Equations (5)–(8) and (20), which takes into account the binding equilibrium (curves in Figure 4a) lead to consistent *cmc* values which depend much less on the dye used (diamonds in Figure 4d).

It is important to note that methods that define the *cmc* at the maximum of the first derivative or at the zero-crossing of the second derivative (or other similar criteria) of the fluorescence intensity will fail to report the correct *cmc* due to the unavoidable influence of the binding equilibrium, even for highly hydrophobic dyes such as perylene or pyrene. Panels (b) and (c) of Figure 4 show the first and second derivatives of the fluorescence intensities of panel (a). Neither the maxima nor the zero-crossings coincide with the *cmc*, nor will higher derivatives or other criteria defined directly based on these intensities.

#### 3.4.1. Steady-State and Time Resolved Fluorescence of Pyrene

Pyrene has been used since more than 50 years as fluorescent probe for microheterogeneous systems [52,53,54,55]. The sensitivity of the pyrene fluorescence to the solvent polarity is widely used for the determination of the *cmc* of micellar systems.

The combination of the surfactant concentration model with a systematic description of the underlying photophysical processes allowed us to reproduce with high accuracy the steady-state and the time-resolved fluorescence intensity of pyrene in surfactant solutions near the *cmc*, both in the monomer and in the excimer emission bands [25].

For values of *r* < 0.4 we presented a simplified equation for the intensity ratio $SR_{m}^{\left(2,1\right)}=I_{m}^{\left(2\right)}/I_{m}^{\left(1\right)}$ at two wavelengths in the monomer bands, for example the *I_I_*/*I_III_* ratio at 372 nm and 384 nm, respectively (“py-scale” [56,57]) with the spectral ratios of free pyrene, $SR_{f}^{\left(2,1\right)}$ at ${\left[S\right]}_{0}=0$, and of bound pyrene, $SR_{b}^{\left(2,1\right)}$ at ${\left[S\right]}_{0}\gg cmc$:

(21) $$SR_{m}^{\left(2,1\right)}\approx \frac{SR_{f}^{\left(2,1\right)}+SR_{b}^{\left(2,1\right)}c[M]}{1+c[M]}$$

The constant $c=q_{bm,f}^{\left(1\right)}K(1+K_{q}\cdot cmc)$ depends on the binding equilibrium constant *K*, the surfactant-dye quenching constant *K_q_*, the brightness ratio *q* between the fluorescence intensities of free and bound monomer and the *cmc*. More detailed equations, also for the monomer-excimer ratios and for time resolved intensities are given in [25].

**Figure 5:**
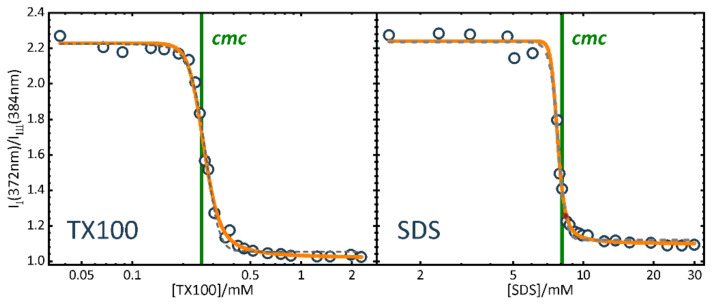
Experimental data of the spectral ratios *I_I_*/*I_III_* of pyrene in TX100 (left) and SDS (right) solutions at 298 K. Orange continuous line: Fit with the concentration model Equation (21) and the parameter values of Table. Grey dashed line: Fit with the sigmoidal Boltzmann function. (Adapted from [25]).Table 1. Results of global fits to the the spectral ratios I/IIII of pyrene in TX100 and SDS solutions at 298 K shown in Figure 5 with the concentration model Equation (21). (Adapted from [25].)

We could show how sensitively the pyrene fluorescence intensity depends on the binding equilibrium constant *K* between pyrene and micelles, on the rate constant of excimer formation in micelles, and on the pyrene-surfactant quenching. We determined the binding equilibrium constants of pyrene to TX100 and SDS micelles (see Table 1). We demonstrated that for a precise determination of the *cmc* from the pyrene fluorescence intensity, especially from the intensity ratio at two vibronic bands in the monomer emission or from the ratio of excimer to monomer emission intensity, the partition equilibrium of pyrene has to be taken into account [25]. Figure 5 compares the sigmoidal shaped *I_I_*/*I_III_* intensity ratio dependence on surfactant concentration for TX100 and SDS with the position of the *cmc* determined from the surfactant concentration model of Equation (21). The *cmc* values coincide very well with those determined from other properties with this model, *cmc* = 8.099 ± 0.005 mM from the conductivity of SDS (see above) and *cmc* = 0.270 ± 0.002 mM from the UV-absorbance of TX100 (see below). Note however, that these *cmc* values (green vertical line in the figure) do not coincide with any specific characteristic point of the sigmoidal functions and how the relative position changes between both surfactants due to the different binding equilibrium constants *K* of pyrene to SDS and TX100 (Table 1).

The finite width of the transition region provides also a plausible explanation for the changes in the pyrene fluorescence observed below the *cmc*. We found no experimental evidence of premicellar aggregates or a shift of the *cmc* due to the presence of pyrene [25].

### 3.5. UV-Vis Absorbance

Direct UV-Vis absorbance of surfactants can be used in some cases to determine their *cmc*. This is useful in the case of neutral surfactants not accessible to conductivity measurements. However, the typically small changes in the absorption spectra require meticulous measurements and careful data analysis.

The absorbance (*A*) of a surfactant at a given wavelength (*λ*) and total surfactant concentration ([S]_0_) is the sum of the absorbance of monomeric and micellized surfactant molecules, given by the respective molar absorption coefficients $\varepsilon_{1}(\lambda )$ and $\varepsilon_{m}(\lambda )$, the concentrations [S_1_] and [S_m_], and the absorption path length ℓ:

(22) $$A(\lambda ,{\left[S\right]}_{0})=[S_{1}]\cdot \varepsilon_{1}(\lambda )\cdot l+[S_{m}]\cdot \varepsilon_{m}(\lambda )\cdot \ell$$

The absorbance ratio at two wavelengths *λ_a_* and *λ_b_* is then:

(23) $$q_{A}(\lambda ,{\left[S\right]}_{0})=\frac{A(\lambda_{b})}{A(\lambda_{a})}=\frac{\left[S_{1}]\cdot \varepsilon_{1}(\lambda_{b})+[S_{m}]\cdot \varepsilon_{m}(\lambda_{b}\right)}{\left[S_{1}]\cdot \varepsilon_{1}(\lambda_{a})+[S_{m}]\cdot \varepsilon_{m}(\lambda_{a}\right)}=\frac{[S_{1}]\cdot q_{1}+[S_{m}]\cdot q_{m}\cdot q_{a}}{[S_{1}]+[S_{m}]\cdot q_{a}}$$

with absorbance ratios $q_{1}$ and $q_{m}$ of each species at the two wavelengths, and ratio *q_a_* of the two species at wavelength *λ_a_* [24].

The UV-absorbance of the neutral surfactant TX100 shows a small but significant bathochromic (red) shift above the *cmc* accompanied by the appearance of a pronounced shoulder around 285 nm [24]. The plot of the absorbance at 285 nm versus surfactant concentration shows a small change in its slope around the *cmc*, which is not observed at 270 nm.

The plot of the ratio of the absorbance at these two wavelengths shows a strong change above the *cmc* that can be very well fitted by Equation (23) and the concentration model. The value *cmc* = 0.270 ± 0.002 mM and the relative transition width *r* = 0.108 ± 0.008 coincide very well with the values obtained from fluorescent probe molecules using the concentration model [24].

### 3.6. Fluorescence Correlation Spectroscopy

Fluorescence Correlation Spectroscopy (FCS) measures the translational diffusion coefficient of a fluorophore in solution. Dye molecules diffuse much faster freely in water than bound to a micelle. As discussed above, even hydrophobic dyes exchange in a fast dynamic equilibrium between micelles and the surrounding water. Therefore, the observed translational diffusion coefficient $\bar{D}$ of a dye in the presence of micelles is the weighted mean value of that of the free dye, *D_f_*, and that of the bound dye, *D_b_* [24,58,59,60,61]:

(24) $$\bar{D}=X_{f}D_{f}+X_{b}D_{b}=\frac{D_{f}}{1+K\cdot [M]}+\frac{D_{b}\cdot K\cdot [M]}{1+K\cdot [M]}$$

In plots of the mean translational diffusion coefficient $\bar{D}$ against the surfactant concentration the diffusion coefficient is that of free dye ($\bar{D}=D_{f}$) below the *cmc*, but its value decreases above the *cmc* as the dye is progressively incorporated into micelles slowing down its diffusion.

In a previous study we compared the diffusion coefficients of three dyes with increasing hydrophobicity (Rhodamine 123, Coumarine 152 and BODIPY-labelled cholesterol) in the presence of TX100 and obtained very good agreement in the values of the *cmc*, *r* and of the limiting diffusion coefficients of free and bound dyes [24].

### 3.7. Aggregation of Non-Surfactant Systems

**Figure 6:**
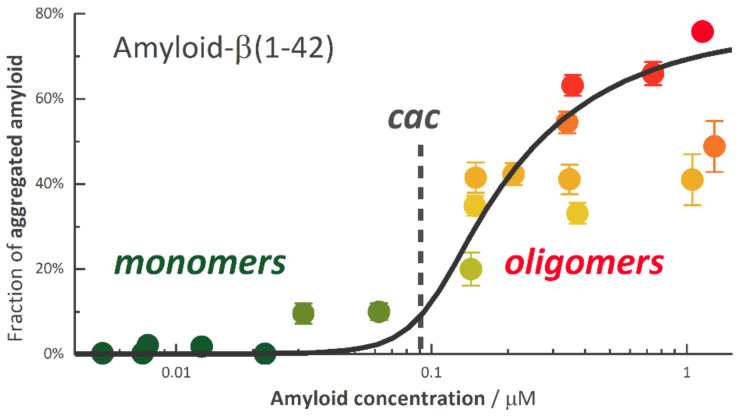
Fraction of aggregated Aβ42, *γ* = [A_g_]/[A] (the degree of aggregation), against the total Aβ42 concentration in solution. The black solid line is the result of a weighted fit of Equation (25) together with the concentration model Equation (7) with a *cac* of 90 nM (vertical dashed line), *r* = 0.36, resulting in a width of the transition region *σ* = *r·cac* = ±33 nM. Note that the weighted fit is mainly defined by the samples with small uncertainties. (Adapted from [62]).

The concentration model also proved to be useful as an empiric model for the definition and estimation of the critical aggregation concentration (*cac*) of Amyloid-β(1–42) (Aβ42), the dominant peptide in amyloid fibrils observed in the brains of patients suffering Alzheimer’s disease. Aβ42 has a very strong tendency to aggregate and to adsorb to surfaces and is notoriously difficult to handle quantitatively. Applying FCS we could determine the fraction *γ* = [A_g_]/[A] of aggregated Aβ42, (A_g_), as a function of total Aβ42 concentration, [A], shown in Figure 6 [62]. For this type of peptide the formation of micelle-like intermediates was reported, so we fitted the fraction with the concentration model of Equation (7), applying the following correspondence:

(25) $$\gamma =\frac{\left[A_{g}\right]}{\left[A\right]}=\frac{\left[S_{m}\right]}{{\left[S\right]}_{0}}=1-\frac{\left[S_{1}\right]}{{\left[S\right]}_{0}}$$

Within the inherent strong uncertainty, the weighted fit reproduced the data well and allowed us to extract the value *cac* = 90 nM of this peptide. Beside the *cac* value, the important result is that Aβ42 undergoes aggregation only when the amount of amyloid monomers exceeds some critical aggregation concentration (*cac*) around 90 nM.

### 3.8. Water-Micelle Partitioning Coefficients from Conductivity and UV-Vis Spectra

Lewandowski and Szymczyk studied the partitioning between water and anionic surfactant micelles of selected anisole and veratrole derivatives, widely used in perfumery [63]. They performed conductivity and density measurements of solutions of the anionic surfactants, sodium lauryl sulfate (SLS, SDS) and sodium lauryl ether sulfate (SLES) in order to determine the volumetric properties of the studied surfactants. The authors calculated values of apparent molar volumes of monomeric and micellized surfactants using the concentration model, Equations (5)–(8). Then they determined the values of the micelle-water partition coefficient, *K_MW_*, for each pair of the perfume-surfactant system by fitting UV spectra of the studied fragrance materials obtained at all concentrations of surfactant to the relation between the absorbance of the solution and the concentration of the micellized surfactant as determined by Equations (5)–(8). The authors obtained excellent fits and could extract valuable information from relatively small changes in the experimental data.

## 4. Influence of Nonlinear Property-Concentration Relationships on the *cmc* Value

The concentration model uses the Phillips condition, Equation (1), to define the *cmc* as the point of maximum change in the gradient of the concentration of monomeric surfactant. As pointed out before, other conditions could have been used. However, once defined, the position of the *cmc* should be independent of the experimental property, the technique or the representation used to determine its value. The *cmc* refers to the surfactant and not to a dependent property.

As long as the property *ϕ* or its representation depends linearly on the surfactant concentration (Equation (26)), the *cmc* defined by Equation (1) also corresponds to the point of maximum change in gradient of the property *ϕ* or its representation. In fact, Phillips originally defined the condition specifically for linear properties (Footnote in [1]).

(26) $$\phi =A[S_{1}]+B[S_{m}]$$

Many properties are linear functions of the surfactant concentration, such as conductivity, Equation (12), NMR-chemical shift, Equation (18), or UV-Vis absorbance, Equation (22). These properties yield in general linear property-concentration curves with a break in the slope at the *cmc*. However, others depend nonlinearly on the concentration: molar conductivity, Equation (13), surface tension, Equation (17), fluorescence intensity of a probe molecule, Equation (20), pyrene band ratio, Equation (21), absorbance ratio, Equation (23), the mean diffusion coefficient of a dye in micellar exchange, Equation (24), and calorimetric data, such as isothermal titration calorimetry (ITC). These properties generally lead to more or less sigmoidal property-concentration curves. For these nonlinear properties, the correspondence between the point of maximum gradient of the concentration and that of the property is lost. Even worse, the position of these curves relative to the *cmc* depends on intrinsic parameters such as the width of the transition region, a binding equilibrium constant (fluorimetry), the adsorption equilibrium constant (ST), etc. The example of Figure 4 illustrates the important influence these parameters have on the property-concentration plot (in this case the binding equilibrium constant in fluorimetry). It is principally an impossible task to define or to determine a coherent *cmc* value from these nonlinear properties as long as the intrinsic parameters are not taken into account. This applies to procedures analysing derivatives of first, second, third or even higher orders of the experimental data, but also to graphical methods looking for intersections of straight lines [10,11,12]. As said before, the critical micellization concentration, *cmc*, is a property of the surfactant concentration itself, not of other derived experimental properties. Properties that depend linearly on the surfactant concentration are well behaved and directly reflect the changes in the concentrations of monomeric and micellized surfactants. Here most methods yield correct and coherent *cmc* values, apart from impurities and other experimental errors. However, those properties that depend nonlinearly on the surfactant concentration distort the surfactant concentration dependence and require a detailed analysis in order to relate their characteristics with the *cmc.*

A general method for the determination of the *cmc* from linear *and* nonlinear properties should be based on a common definition of the *cmc* directly related to the surfactant concentration itself and then derive the experimental properties from this concentration. The concentration model we propose is not the only possible and can still be refined and adapted, but it has so far proven to reproduce excellently several properties, to yield coherent *cmc* values and to contribute to a better understanding of how properties depend on the surfactant concentrations, the *cmc* and the transition width [23,24,64].

The concentration model also allows one to deduce new property-concentration relationships and to validate them experimentally. For example, in order to clarify the nucleation role of the cationic surfactant CTAB, Dvorsky et al. derived an expression for the mathematical dependence of the ZnS nanoparticle radius on the CTAB concentration based on Equation (7) that reproduced the experimental data within a relative uncertainty as low as 0.34% [65].

## 5. Analysis with Sparse Data Points

The concentration model can also be used in those cases where the transition region is not well defined due to the lack of data points. The determination of the *cmc* with the concentration model is robust and mostly independent of the value of the transition width. The uncertainty in the *cmc* value depends more on the overall quality of the data points than on the presence or not of values near the *cmc*.

**Figure 7:**
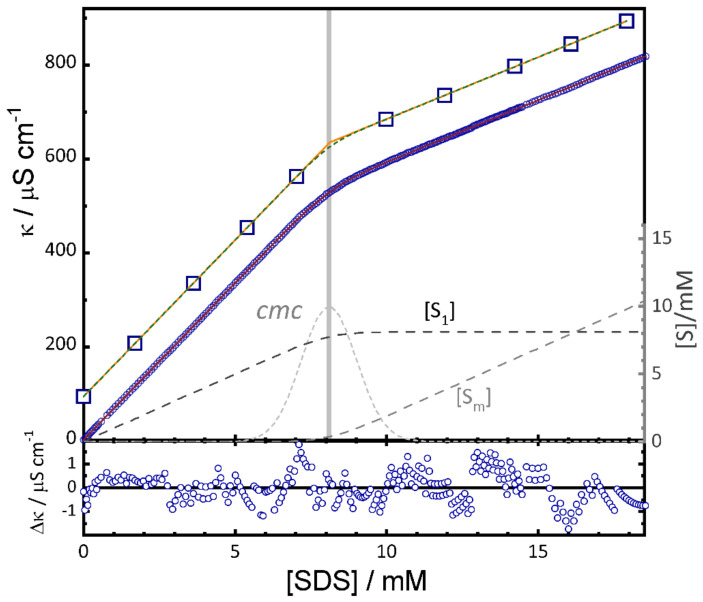
Conductivity of SDS in aqueous solution at 298 K. Full data set: the small circles are the 332 data points. The red solid line behind the circles indicates the fit of model Equation (12) to this data. Reduces data set: the big squares are 10 data points taken from the full data set and shifted upwards for better visibility. Orange continuous line and grey dashed line are fits of Equation (12) with fixed values *r* = 0.1 and 0.001, respectively. Dashed grey lines: concentrations [S_1_] and [S_m_] from Equations (5) and (7). Dotted grey line: second derivative of [S_1_] (Equation (2)). Vertical grey line: *cmc* as given by the fit. Lower panel: residuals of the fit to the full data set. (Adapted with permission from [9]).

In the Figure 7 we compare the fit of a dataset of 332 points of the conductivity of SDS with that of the same data eliminating all but 10 points. The results of the fits are given in Table 2. The fit to all points gives precise values of the *cmc* and the relative transition width already presented before [9]. Of course, the transition width is very poorly defined in the fit to the reduced data set. However, fits with fixed values of *r* = 0.1 or *r* = 0.001 lead within error limits to the same value of the *cmc* as with the full data set. Fixing the value of *r* to a small value effectively determines the *cmc* at the intersection of the two straight lines determined by the slopes *a* and *b*.

## 6. Conclusions

We have shown that the combination of a surfactant concentration model with known equations for the concentration-dependence of different surfactant properties makes it possible to determine the *cmc* and the transition width of surfactant solutions and their mixtures in a systematic and unambiguous way. We revised its application to a variety of properties, such as electrical conductivity, NMR chemical shift, self-diffusion, surface tension, UV-Vis absorption, fluorescence intensity and fluorescence correlation. The model equations we propose yield for different properties coherent *cmc* values for a given type of surfactant. It also opens the way to develop new models for property-concentration relationships or to detect and overcome the limitations of existing ones. The concentration model allows one to extract *cmc* values from nonlinear property-concentration relationships, such as ST or fluorescent probes, which are consistent with those determined from linear properties and with the proper definition of the *cmc* based on the surfactant concentration. The model yields also an objective measure of the width of the transition interval around the *cmc*. Its dependence on composition or temperature or other parameters can yield valuable information not exploited so far. The determination of the *cmc* from nonlinear fits, even global ones, yields not only objective and consistent values, but also information about their statistical uncertainty of great relevance for the correct interpretation of observed variations.

## Figures and Tables

**Table 1:** Results of global fits to the the spectral ratios *I_I_*/*I_III_* of pyrene in TX100 and SDS solutions at 298 K shown in Figure 5 with the concentration model Equation (21). (Adapted from [25]).

	TX100	SDS
*cmc*/10^−3^ mol L^−1^	0.275 ± 0.005	8.1 ± 0.1
*r*	0.16 ± 0.01	0.07 ± 0.03
*K*/10^3^ mol L^−1^	3300 ± 200	190 ± 40
*K_q_*/10^3^ mol L^−1^	2.2 ± 0.2	0.23 ± 0.03
$SR_{f}^{\left(2,1\right)}$/$SR_{b}^{\left(2,1\right)}$/$q_{b,f}^{\left(1\right)}$ ^[a]^	0.45/0.98/1.5	0.44/0.91/1.4

^[a]^ *λ*_1_ = 372 nm (peak I), *λ*_2_ = 384 nm (peak III).

**Table 2:** Results of the fit with the concentration model Equations (7) and (12) to the data of SDS in aqueous solution at 25 °C shown in Figure 7.

Data	*cmc*/mM	*r*	*a* ^(1)^	*b* ^(1)^
all points	8.099 ± 0.005	0.112 ± 0.001	66.74 ± 0.03	26.43 ± 0.01
10 points	8.12 ± 0.02	0.08 ± 0.03	66.6 ± 0.1	26.37 ± 0.08
10 points	8.10 ± 0.02	0.1 fixed	66.75 ± 0.09	26.35 ± 0.08
10 points	8.12 ± 0.02	0.001 fixed	66.60 ± 0.09	26.37 ± 0.07

^(1)^ Unit: μS cm^−1^ mM^−1^.

## Data Availability

The data presented in Figure 2, Figure 3 and Figure A1 are available in [37] and that of Figure A2 in the SI of [31]. Fitting functions of the concentration model are available on the web page of the authors.

## Appendix A

**Table A1:** Polynomial fit $cmc=a+b\cdot T+c\cdot T^{2}$ of the *cmc* values of IMP and ClMP hydrochloride at different temperatures obtained from global fits of the data of Figure 2 and Figure A1.

*cmc*/mol kg^−1^	*a*/mol kg^−1^	*b*/mol kg^−1^ K^−1^	*c*/mol kg^−1^ K^−2^
ClMP	0.30 ± 0.04	(−2.2 ± 0.2) × 10^−3^	(4.3 ± 0.4) × 10^−6^
IMP	0.29 ± 0.03	(−2.1 ± 0.2) × 10^−3^	(4.2 ± 0.4) × 10^−6^
